# Modulation of colony stimulating factor release and apoptosis in human colon cancer cells by anticancer drugs

**DOI:** 10.1038/sj.bjc.6600240

**Published:** 2002-04-22

**Authors:** S Calatayud, T D Warner, J A Mitchell

**Affiliations:** Unit of Critical Care, The Royal Brompton and Harefield N.H.S. Trust, Imperial College School of Medicine, Sydney Street, London SW3 6NP, UK; The William Harvey Research Institute, St. Bartholomew's and the Royal London School of Medicine and Dentistry, Charterhouse Square, London EC1M 6BQ, UK

**Keywords:** colonic cells, apoptosis, colony stimulating factors, anticancer drugs

## Abstract

Modulation of the immune response against tumour cells is emerging as a valuable approach for cancer treatment. Some experimental studies have shown that secretion of colony stimulating factors by cancer cells reduces their tumorigenicity and increases their immunogenicity probably by promoting the cytolitic and antigen presenting activities of leukocytes. We have observed that human colon cancer cells (HT-29) are able to secrete granulocyte-macrophage-colony stimulating factor, granulocyte-colony stimulating factor and macrophage-colony stimulating factor when stimulated with cytokines (IL-1β and TNF-α). In this study we assessed, for the first time, the effects of several anticancer drugs on colony stimulating factor release or apoptosis in HT-29 cells. Cytokine-induced release of granulocyte-macrophage-colony stimulating factor, granulocyte-colony stimulating factor and macrophage-colony stimulating factor was significantly increased by cisplatin and 6-mercaptopurine. Taxol only increased macrophage-colony stimulating factor release while reduced that of granulocyte-colony stimulating factor. No changes in colony stimulating factor secretion were observed after treatment with methotrexate. Only cisplatin and taxol induced apoptosis in these cells. Secretion of colony stimulating factors by colon cancer cells may contribute to the immune host response against them. Anticancer drugs such as cisplatin and 6-mercaptopurine increase colony stimulating factor secretion by cytokine stimulated cancer cells probably through mechanisms different to those leading to cell apoptosis, an effect that may contribute to their anti-neoplasic action.

*British Journal of Cancer* (2002) **86**, 1316–1321. DOI: 10.1038/sj/bjc/6600240 www.bjcancer.com

© 2002 Cancer Research UK

## 

Treatment of cancer widely relies on the use of cytotoxic drugs. Anticancer drugs interfere with different steps involved in the processes of cell division and so damage cancerous cells. In many instances these drug effects are now known to be via influences on the apoptotic pathway. In addition, there is now an increasing interest in manipulating the body's immune response against cancer cells. Indeed, genetic therapies aimed at increasing the secretion of certain cytokines by cancer cells seem to have potential as a mode of increasing the host response against tumour progression (Dranoff and Mulligan, 1995; Ettinghausen and Rosenberg, 1995). In particular, increasing the secretion of the colony stimulating factors (CSFs) granulocyte-macrophage-CSF (GM-CSF) or granulocyte-CSF (G-CSF) by colon adenocarcinoma, melanoma, lung or renal carcinoma, fibrosarcoma, B-cell lymphoma or acute myeloid leukemic cells prevents the formation of tumours by these cells and results in the elimination of pre-established cancer in mice (Colombo *et al*, 1991; Dranoff *et al*, 1993; Armstrong *et al*, 1996; Levitsky *et al*, 1996; Dunussi-Joannopoulos *et al*, 1998).

In addition to their recently noted effects on cancer cells, CSFs such as G-CSF, GM-CSF or macrophage-CSF (M-CSF) have multiple biological activities. These biological activities include the ability to stimulate the proliferation and differentiation of multipotential bone marrow progenitor cells into mature granulocytes and macrophages (Fleischmann *et al*, 1986). There is evidence that CSFs stimulate the phagocytic and cytocidal activity of macrophages (Handman and Burgess, 1979; Ho *et al*, 1990) and promote monocyte and neutrophil antibody-dependent cellular cytotoxicity (Kushner and Cheung, 1989; Liesveld *et al*, 1991). They may also play a major role in the maturation of dendritic cells into potent activators of resting T cells (Morrissey *et al*, 1987). In fact, the beneficial effect of CSF secretion by cancer cells appears to be due to the increased recruitment and activity of phagocytic cells as well as to an enhancement of the antigen presentation activity and T-cell mediated cytotoxicity.

We have observed that human colon cancer cells are able to secrete G-CSF, M-CSF and GM-CSF (Calatayud *et al*, 2001). However, the mechanisms modulating these releases are incompletely understood. Moreover, the potential effects of anti-cancer drugs on CSF release by these cells have not been investigated. Thus, here we have assessed the influences of a number of different anticancer drugs on the release of G-CSF, M-CSF and GM-CSF. Additionally, we have evaluated whether the effects of these drugs on CSF secretion depend upon the induction of cell apoptosis.

## MATERIALS AND METHODS

### Cell culture

HT-29 cells (Human Caucasian Colon adenocarcinoma Grade II) were obtained from the European Collection of Animal Cell Culture. Cells were seeded at a density of 2×10^4^ per well in 96-well plates, cultured in McCoy's medium with 2 mmol l^−1^ glutamine and 10% foetal calf serum (37°C, 5% CO_2_) and grown in monolayers to confluency. Cells were serum starved for 24 h prior to use. In preliminary studies, cells were stimulated with different combinations of cytokines (Table 1

**Table 1:**
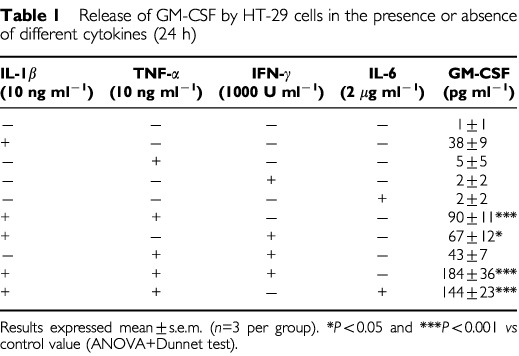
Release of GM-CSF by HT-29 cells in the presence or absence of different cytokines (24 h)

) and interleukin-1ß (IL-1β, 10 ng ml^−1^, Roche) plus tumour necrosis factor α (TNF-α,10 ng ml^−1^, Roche) was selected as the optimal treatment for a consistent release of CSFs. The effects of these cytokines on CSF secretion were evaluated in the presence or absence of different concentrations (10^−11^–10^−4^ M) of 6-mercaptopurine (6-MP), cisplatin, taxol, colchicine or methotrexate (all from Sigma). In some experiments, cells were treated with cycloheximide (10^−6^ M) at the time of, or 6 h before, cytokine addition. Medium was removed 24 h after cytokine treatment for the measurement of CSFs. Each treatment was tested in triplicate, on a minimum of three separate occasions.

### Measurement of CSFs

The concentrations of GM-CSF, G-CSF and M-CSF were measured in cell culture supernatant using specific ELISAs constructed from commercially available components. In brief, 96-well plates were coated with the correspondent capture antibodies (rat anti human GM-CSF, G-CSF or M-CSF, R&D systems). Following 1 h blocking (1% BSA, 5% sucrose and 0.05% NaN_3_ in PBS, 100 μl well^−1^), 100 μl of standard or undiluted cell culture supernatant was added and incubation continued for a further 2 h. The wells were subsequently incubated with the respective detection antibodies (rat anti human GM-CSF, G-CSF or M-CSF, R&D systems, 2 h) and streptavidin peroxidase (Sigma, 30 min). Finally, 100 μl of substrate solution (1:1 mixture of tetramethylbenzidine and hydrogen peroxide) was added and the reaction stopped 30 min later by addition of 50 μl of 1 N H_2_SO_4_. Absorbance was then read at 450 nm (λ correction 550 nm) and the concentrations of CSFs calculated from the standard curves.

### Measurement of apoptosis

At the end of each treatment the level of apoptosis was evaluated. Apoptosis was measured by the degree of cytoplasmic histone associated DNA fragments (mono- and oligonucleosomes) by ELISA (Programmed Cell Death Detection ELISA, Roche) according to the manufacturer's recommendations. Briefly, the medium was removed and the cells lysed in 200 μl of the lysis buffer provided. The lysate was centrifuged and 20 μl of supernatant added to a streptavidin-coated microtiter plate with a mixture of mouse biotin-labelled anti-histone and mouse peroxidase-conjugated anti-DNA. After 2 h-incubation period, the plate was washed and the retained peroxidase determined photometrically with ABTS (2,2′-azino-di-[3-ethylbenzthiazoline sulphonate]) as a substrate. Optical density was measured at 405 nm (λ correction 492 nm).

### Measurement of viable cell number (MTT assay)

Cellular respiration was assessed by mitochondrial-dependent reduction of 3-[4,5-dimethylthiazol-2-yl]-2,5-diphenyltetrazolium bromide (MTT, Sigma) to formazan. At the end of the experiment, medium was removed from the cells and replaced with 100 μl of warm (37°C) medium containing 0.2 mg ml^−1^ of MTT. After incubation for a further 30 min at 37°C the medium containing the MTT was removed and the cells dissolved in 100 μl dimethyl sulphoxide (DMSO). The extent of MTT conversion to formazan was quantified by measurement of optical density at 550 nm with a wavelength correction of 650 nm.

## RESULTS

### CSF production by HT-29 cells

**Figure 1:**
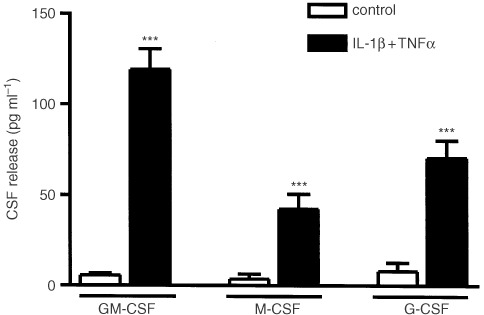
Release of GM-CSF, M-CSF and G-CSF by HT-29 cells in the presence or absence of IL-1β and TNF-α (both 10 ng ml^−1^, 24 h). Results expressed as mean±s.e.m. (*n*=20 per group). ***P<0.001 *vs* respective control value (2-tailed Student's *t*-test).

In the absence of cytokines, HT-29 cells released low or undetectable levels of GM-CSF, G-CSF or M-CSF. However, these cells can be stimulated with common pro-inflammatory cytokines to secrete CSFs (Table 1). Preliminary studies aimed to select the optimal treatment for a consistent release of CSFs showed an increased GM-CSF secretion after a 24 h-incubation period with IL-1β (10 ng ml^−1^). This response was significantly enhanced by co-treatment with TNF-α at a concentration devoid of any secretory effect *per se* (10 ng ml^−1^). The combined action of IL-1β plus TNF-α was further enhanced by interferon-γ (1000 U ml^−1^) or interleukin-6 (2 μg ml^−1^) (Table 1) although in these cases, significant reductions in the number of viable cells were observed (data not shown). Secretion of G-CSF and M-CSF was also significantly increased by IL-1β plus TNF-α (10 ng ml^−1^ both, 24 h) (Figure 1

) and thus, this combination of cytokines was selected for cell stimulation.

**Figure 2:**
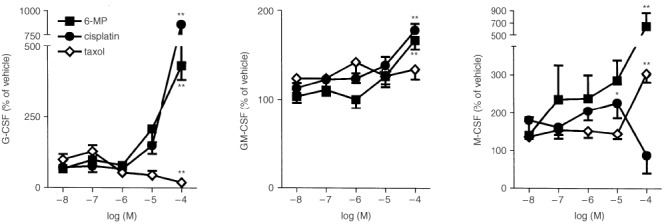
Effects of 6-MP, cisplatin and taxol on the release of G-CSF, GM-CSF or M-CSF by cytokine stimulated HT-29 cells (IL-1β and TNF-α, both 10 ng ml^−1^, 24 h). Results expressed as mean±s.e.m (*n*=5 per group). *P<0.05, **P<0.01 *vs* value in the respective vehicle-treated cells (ANOVA+Dunnet test).

Cytokine-induced release of CSFs was tested in the presence of several anti-cancer drugs with different mechanisms for their anti-neoplasic action. Incubation with the purine analogue 6-MP or the DNA-damaging agent cisplatin (10^−6^–10^−4^ M both) induced significant increases in GM-CSF, G-CSF and M-CSF secretion by cytokine treated HT-29 cells (Figure 2

). By contrast, the inhibitor of microtubule depolymerisation taxol produced divergent effects increasing M-CSF secretion, reducing G-CSF secretion and leaving GM-CSF secretion unchanged (Figure 2). Finally, the dihydrofolate reductase inhibitor methotrexate had no effects upon the production of any of the CSFs (data not shown).

**Figure 3:**
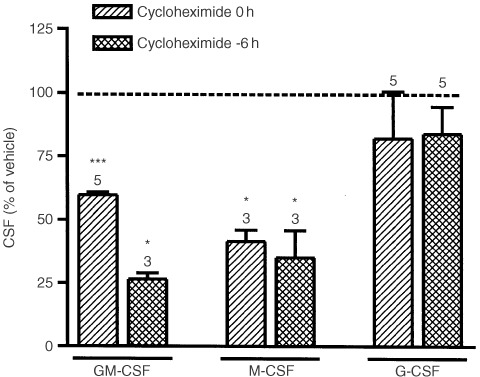
Effects of cycloheximide (10^−6^ M) on the release of GM-CSF, M-CSF or G-CSF by cytokine stimulated HT-29 cells (IL-1β and TNF-α, both 10 ng ml^−1^, 24 h). Cycloheximide was added to the medium at the same time or 6 h before cytokines. Results expressed as mean±s.e.m. of the number of experiments shown above each column. *P<0.05 and ***P<0.001 *vs* value in the respective vehicle-treated cells (2-tailed Student's *t*-test).

**Figure 4:**
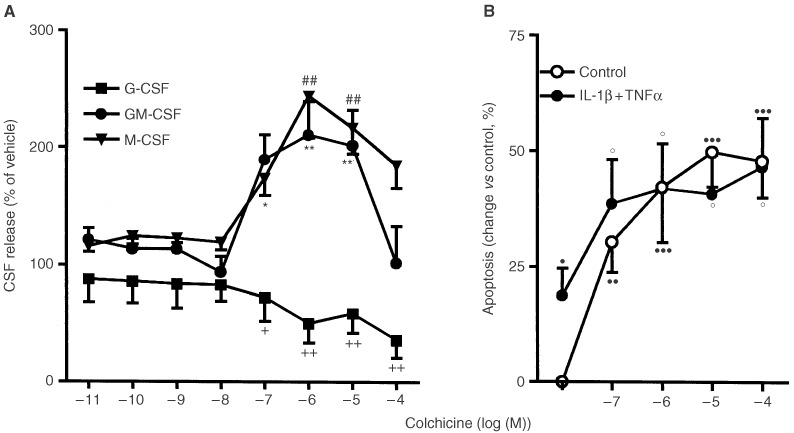
Effects of colchicine on (**A**) G-CSF, GM-CSF or M-CSF release by cytokine stimulated HT-29 cells (IL-1β and TNF-α, both 10 ng ml^−1^, 24 h) and (**B**) HT-29 cell apoptosis in the presence or absence of these cytokines. Results expressed as mean±s.e.m. (*n*=3 per group). *P<0.05, **P<0.01, ^+^*P*<0.05, ^++^*P*<0.01 and ^##^*P*<0.01 *vs* value in the respective vehicle-treated cells; ^•^*P*<0.05, ^••^*P*<0.01 ^•••^*P*<0.001 *vs* control vehicle-treated cells; °*P*<0.05 *vs* cytokine+vehicle-treated cells (ANOVA + Dunnet test).

In order to clarify the mechanism for the release of CSFs by HT-29 cells after cytokine treatment, cells were incubated with an inhibitor of protein synthesis (cycloheximide 10^−6^ M) or colchicine (10^−11^–10^−4^ M), which induces microtubule depolymerisation. Pre-treatment or co-treatment with cycloheximide inhibited cytokine-induced release of GM-CSF and M-CSF but not that of G-CSF (Figure 3

). Colchicine (10^−7^–10^−4^ M) increased GM-CSF and M-CSF secretion whilst it decreased that of G-CSF (Figure 4A

).

### HT-29 cell apoptosis and viability

**Figure 5:**
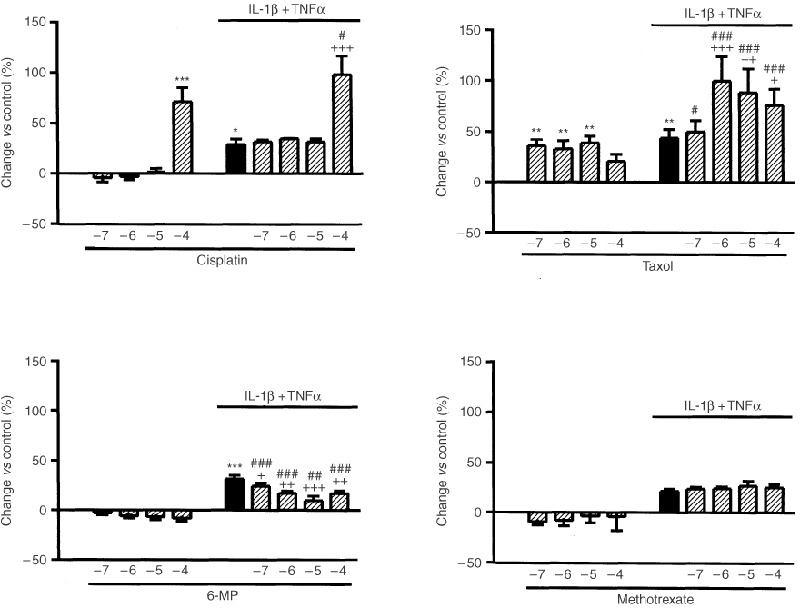
Effects of cisplatin, taxol, 6-MP and methotrexate on HT-29 cell apoptosis in the presence or absence of IL-1β and TNF-α (both 10 ng ml^−1^, 24 h). Results expressed as mean±s.e.m. (*n*=3 per group). *P<0.05, **P<0.01 and ***P<0.001 *vs* control conditions, ^+^*P*<0.05, ^++^*P*<0.01 and ^+++^*P*<0.001 *vs* cytokine treated cells, ^#^*P*<0.05, ^##^*P*<0.01 and ^###^*P*<0.001 *vs* same drug concentration in non-cytokine treated cells (ANOVA+Tukey test).

Cisplatin and taxol significantly increased apoptosis of HT-29 cells 24 h after treatment. Cell apoptosis was also augmented by IL-1β plus TNF-α and this effect was additive to those of cisplatin or taxol (Figure 5

). The increase in apoptosis of HT-29 cells observed after incubation with cytokines plus cytotoxic drugs was accompanied by reductions in the number of viable cells (Table 2

**Table 2:**
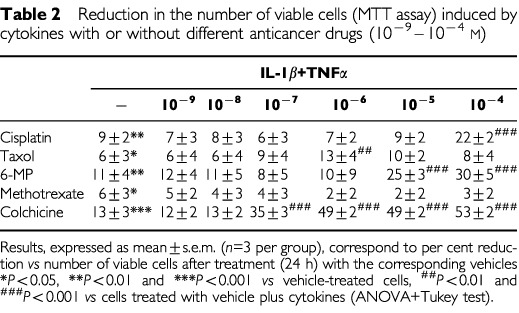
Reduction in the number of viable cells (MTT assay) induced by cytokines with or without different anticancer drugs (10^−9^–10^−4^ M)

). 6-MP or methotrexate did not induce HT-29 cell apoptosis in control conditions or in the presence of cytokines at the time point used (Figure 5). However, when 6-MP was given to cells together with cytokines a reduction in viable cell number was seen (Table 2). HT-29 cell apoptosis was increased in the presence of colchicine and this effect was not additive to that of the cytokines (Figure 4B).

Statistical analysis of the data showed no correlation between the increases in apoptosis or the reductions in cell viability induced by these drugs and their effects on the production of GM-CSF, G-CSF or M-CSF (data not shown).

## DISCUSSION

The functional effects of granulocytes, lymphocytes, and macrophages are important in patients with malignancies because of the abilities of these cells to exhibit antitumour activities. Because of these abilities, different immunotherapy strategies have been assayed for cancer treatment. In experimental studies tumour regression follows increases in the local release of different cytokines that would induce the recruitment and activation of immune cells (Dranoff and Mulligan, 1995; Ettinghausen and Rosenberg, 1995). Indeed, CSFs have been shown to be amongst the most potent immune response inducers in this setting (Colombo *et al*, 1991; Dranoff *et al*, 1993; Armstrong *et al*, 1996; Levitsky *et al*, 1996; Dunussi-Joannopoulos *et al*, 1998).

In the present study we confirm our previous observations that human colon cancer epithelial cells have the ability to produce GM-CSF, G-CSF and M-CSF in response to cytokines commonly found in the inflammatory responses (IL-1β and TNF-α), including those that accompany tumour formation. Moreover, we have shown, for the first time, that the secretion of CSFs by colon cancer cells stimulated with these cytokines is modified by different anticancer drugs.

In our study the purine analogue 6-MP and the DNA-damaging agent cisplatin acted similarly. They both increased the secretion of all three CSF by cytokine-stimulated HT-29 cells. Colchicine, which binds to a site on ß-tubulin and causes microtubule depolymerisation, also increased the release of GM-CSF and M-CSF, but decreased the release of G-CSF. Taxol, which inhibits microtubule depolymerisation, similarly increased M-CSF but decreased G-CSF production and did not modify GM-CSF secretion. By contrast methotrexate, an inhibitor of dihydrofolate reductase, failed to affect the release of any CSF.

The aim of antineoplasic treatment with these drugs is to impair mitosis in cancer cells, block progression through the cell cycle, and so promote apoptosis. However, cancer cells often develop resistance to chemotherapy, which may be drug-specific or mediated by more general mechanisms such as the mutation of p53 suppressor oncogene observed in HT-29 cells (Shao *et al*, 1996). Of the drugs tested in the present study only cisplatin, taxol and colchicine induced apoptosis in HT-29 cells and no correlations between changes in CSF secretion and induction of apoptosis were observed in any case. For instance, similar effects on CSF secretion were obtained with 6-MP and cisplatin, and only cisplatin induced apoptosis in HT-29 cells.

Taxol and colchicine both modify microtubule formation and both drugs reduced G-CSF secretion. However, the effects of taxol and colchicine on GM-CSF and M-CSF release were different. Colchicine induced a significant increase in the release of both cytokines. By contrast, taxol only increased M-CSF secretion and only at concentrations that were much higher than those necessary to modify microtubule function. Experiments performed in the presence of cycloheximide suggested that the cytokine-induced release of GM-CSF and M-CSF by HT-29 cells depends on *de novo* protein synthesis, while G-CSF secretion does not. Taken together these findings suggest that G-CSF secretion in response to cytokines occurs via a tubulin driven release of pre-formed G-CSF.

Previous studies have shown that cisplatin and taxol modify the function of immune cells. For example, treatment with these drugs induces an increase in the secretion of pro-inflammatory cytokines by leukocytes, especially monocytes and macrophages, which is usually accompanied by an enhanced activity and cytotoxicity against cancer cells (Bogdan and Ding, 1992; Gan *et al*, 1992; Suresh and Sodhi, 2001). The present results extend our knowledge of the immunomodulatory effects of cisplatin and taxol, showing that these drugs regulate the synthesis and secretion of CSFs by cytokine-stimulated cancer cells. More unexpected were the results obtained with 6-MP, a drug that besides its action as antineoplasic agent is used in the treatment of chronic inflammatory circumstances such as Crohn's disease. Its utility in Crohn's disease appears due to its immunosupressive effects, although it has also been demonstrated to have some anti-inflammatory properties (Goldstein, 1987). However, treatment of HT-29 cells with 6-MP induced a significant increase in the cytokine-induced release of GM-CSF, G-CSF and M-CSF.

Secretion of CSFs by cancer cells has important consequences for tumour progression. For instance, in murine models of colon adenocarcinoma, melanoma or acute myeloid leukaemia transfection of cancer cells with genes to overexpress G-CSF or GM-CSF results in reduced tumorigenicity (Colombo *et al*, 1991; Armstrong *et al*, 1996; Dunussi-Joannopoulos *et al*, 1998). Additionally, injection of irradiated tumour cells expressing murine GM-CSF produces potent, specific and long-lasting anti-tumour immunity and even induces regression of pre-established cancer (Dranoff and Mulligan, 1995; Armstrong *et al*, 1996; Levitsky *et al*, 1996; Dunussi-Joannopoulos *et al*, 1998). Likewise, some preliminary clinical trials testing the therapeutic potential of systemic GM-CSF administration have produced encouraging results (Armitage, 1998). Rejection of CSF-secreting cancer cells appears to be because they increase the recruitment and activity of phagocytic cells, while the specific immunisation is probably related to the enhanced antigen presentation activity and T-cell mediated cytotoxicity. Increased levels of CSFs following systemic administration may also have beneficial effects by helping to reverse the defective antigen presentation and tumoricidal activities that are found in mononuclear phagocytes isolated from cancer patients (Kleinerman *et al*, 1980). However, a localised release of these cytokines would have extra benefits by attracting the inflammatory cells to the tumour and facilitating their contact with the cancer cells, as pointed out by some experimental studies (Colombo *et al*, 1991; Armstrong *et al*, 1996). Our findings suggest that changes in the secretion of CSFs by cancer cells themselves after treatment with anticancer drugs, may be of significance for the immune response against those cells. This may be particularly important when considering the mechanism of action of drugs such as cisplatin and 6-MP. Thus, we suggest that treatment with these drugs may modulate the host immune response against cancer cells and this could contribute to their anti-neoplasic action. New investigations are required to analyse whether these effects take place *in vivo* and whether anticancer drugs act preferentially in cancer cells.
